# The Myopectineal Orifice: A Study of Thai Cadavers

**DOI:** 10.3389/fsurg.2022.843344

**Published:** 2022-04-05

**Authors:** Amarit Tansawet, Thanakorn Rodsakan, Wisit Kasetsermwiriya, Sopon Lerdsirisopon, Suphakarn Techapongsatorn

**Affiliations:** Department of Surgery, Faculty of Medicine Vajira Hospital, Navamindradhiraj University, Bangkok, Thailand

**Keywords:** myopectineal orifice, dimension, inguinal hernia, cadaver, Thai

## Abstract

**Objective:**

This study aimed to determine the myopectineal orifice size measured in Thai human cadavers.

**Materials and Methods:**

A total of 30 human cadavers, comprising 55 groins, were assessed. Myopectineal orifices (MPOs) were measured in two dimensions: height from the lower border of the conjoined tendon to the upper border of the pectineal ligament and width from the lateral border of pubic tubercle to the medial border of the iliopsoas muscle.

**Results:**

The mean MPO size is 7.13 + 0.14 cm in width and 6.66 + 0.32 m in height. The mean width and height in male cadavers are 7.16 + 0.14 and 6.84 + 0.27 cm, respectively. The mean width and height in female cadavers are 7.09 + 0.12 and 6.45 + 0.24 cm, respectively. The mean MPO area is 37.26 ± 0.027 cm^2^, compared with the area of mesh graft 10 cm × 15 cm, 150 cm^2^. Although the shrinkage of cadaveric tissue and mesh size were adjusted, which were 39.56 ± 0.029 and 81 cm^2^, respectively, they were found to be sufficient for the mean MPO area. It was found that the mesh size was sufficient for the mean MPO area.

**Conclusion:**

A mesh size of 10 cm × 15 cm is found to be the appropriate size to cover the MPO among Thais.

## Introduction

Inguinal hernia is a common problem worldwide and more commonly occurs in men comparedwith women with bimodal distribution in two age groups: during childhood and aged older than 60 years (1). Although many patients with inguinal hernia are asymptomatic or only suffer minimal symptoms if an intestinal obstruction, incarceration, or strangulation occurs, its prognosis may be changed to critical and potentially result in fatal complications (1). According to the recommendation from the International guidelines for groin hernia management, surgery is indicated for symptomatic inguinal hernia (2). However, the major adverse events after inguinal hernia repair were hernia recurrence and chronic groin pain, which were concerns for both the patient and the surgeon. The basic knowledge of inguinal anatomy, mesh biomechanics, and surgeons' experience are essential in surgical repairs that support inguinal hernia repair to be effective and reduce complications. Inguinal anatomy reveals hernia protrusion through an anatomic defect in the inguinal area: direct inguinal hernia *via* Hasselbach's triangle, indirect inguinal hernia at the deep inguinal ring, and femoral hernia at the femoral canal. This triple area is well known as a myopectineal orifice (MPO), named by Henri Fruchaud in 1956 (3, 4). Because a mesh can reinforce MPO to become healthier by integration to the inguinal tissue, mesh-based repair has become the standard surgical technique for symptomatic inguinal hernia (2).

Therefore, proper hernia repair is determined by the mesh's size selection to cover the MPO. In a cadaveric-based study in Germany, Wolloscheck et al. tried to measure the diameter of the MPO, and its dimensions were 7.8 cm × 6.5 cm × 4.5 cm (5). However, the effect of ethnicity on body contour may affect the different sizes of groin anatomy. Therefore, this study aims to demonstrate the inguinal anatomy and calculate the size of MPO in Thai cadaveric, which in turn reflects the proper mesh size.

## Materials and Methods

The Vajira institutional review board approved the study protocol, and authors officially contacted the Department of Anatomy, Faculty of Medicine, King Chulalongkorn Memorial Hospital, Thailand, for the evaluation of inguinal anatomy on Thai cadaver donates.

**Figure 1:**
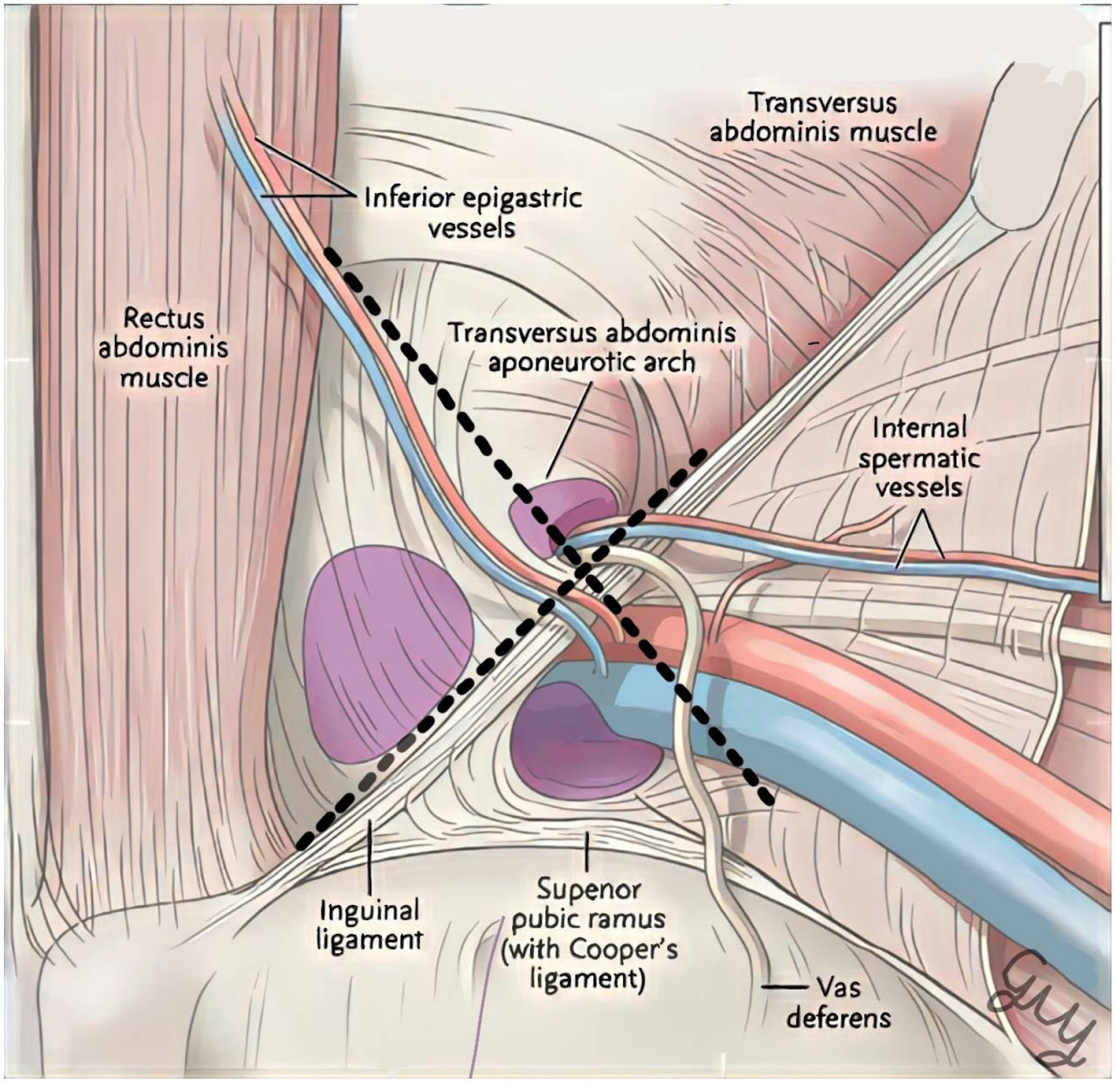
Dimension of the myopectineal orifice.

All cadavers were preserved with 10% formalin and kept for anatomical study. The inclusion criteria for this study is a cadaver that did not undergo inguinal or pelvic surgery. MPO dimension is indicated as the height from the lower border of the conjoined tendon to the upper border of the pectineal ligament and width from the lateral border of pubic tubercle to the medial border of the iliopsoas muscle. Two individual surgeons performed the measurement process, and a Vernier caliper (Kendo, Saame Tools (Shanghai) Import & Export Co., Ltd.) was used to record measurements in centimeters (Figure 1).

**Figure 2:**
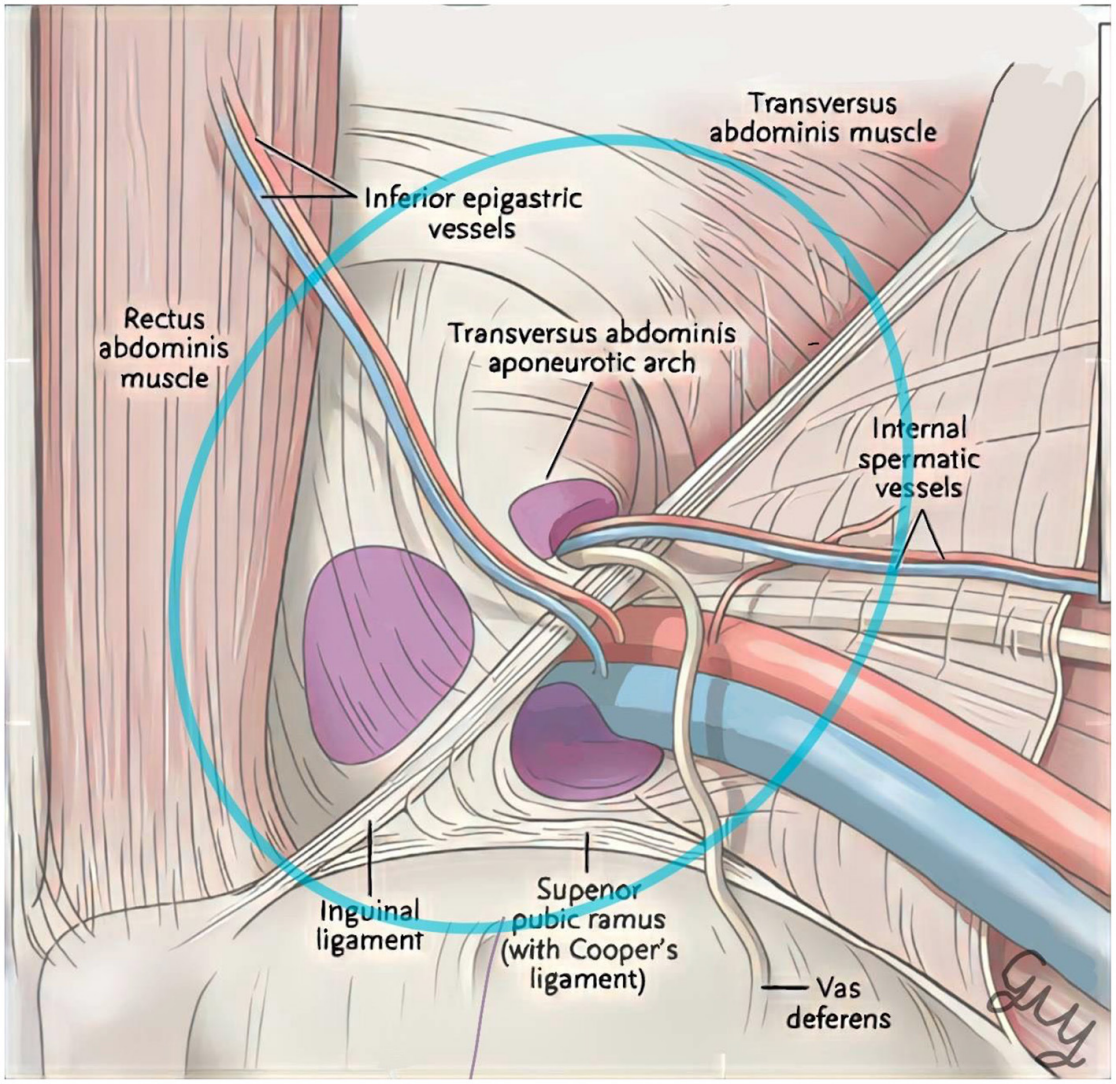
The myopectineal orifice area calculation.

The average height and width of the MPO, with their *SD*s, were used for further Monte-Carlo simulation with 5,000 replications. Then, the MPO areas were calculated using the equation of the ellipse, a × b × π (Figure 2), for each pair of replicated MPO height and width. A 3% of the calculated MPO area were added to compensate cadaveric tissue shrinkage (5).

A 10 cm × 15 cm mesh was selected as a reference regarding hernia repair guidelines (2). By taking into account of percentage of mesh shrinkage ranging from 4.2 to 46% (6–10), mesh areas were calculated and compared with the simulated MPOs. The probability of incomplete MPO coverage (i.e., MPO area larger than mesh area) was estimated accordingly.

## Results

A total of 55 groins from 30 cadavers (16 men and 14 women) were measured and included in this study (5 groins underwent previous tissue dissection and were excluded because of the possibility of inaccurate MPO measurement). Mean age and body mass index (BMI) were 61.88 ± 15.44 years and 23.91 ± 2.77 kg/m^2^, respectively (Table 1).

**Table 1:** The baseline characteristic of cadavers.

Number of cadavers	30
Number of groin measurement	55
Sex–male:female	16:14
Age (mean ± SD)	61.88 ± 15.44
- Male	60.10 ± 12.35
- Female	70.20 ± 12.12
BMI (mean ± SD)	23.91 ± 2.77
- Male	23.30 ± 2.08
- Female	25.14 ± 3.60
Cause of deatd	
- Elderly	5
- Cardiovascular disease	12
- Pulmonary disease	7
- Cancer	4
- Trauma	2
Mean MPO width (cm)	7.13 ± 0.14
Mean MPO height (cm)	6.66 + 0.32

**Figure 3:**
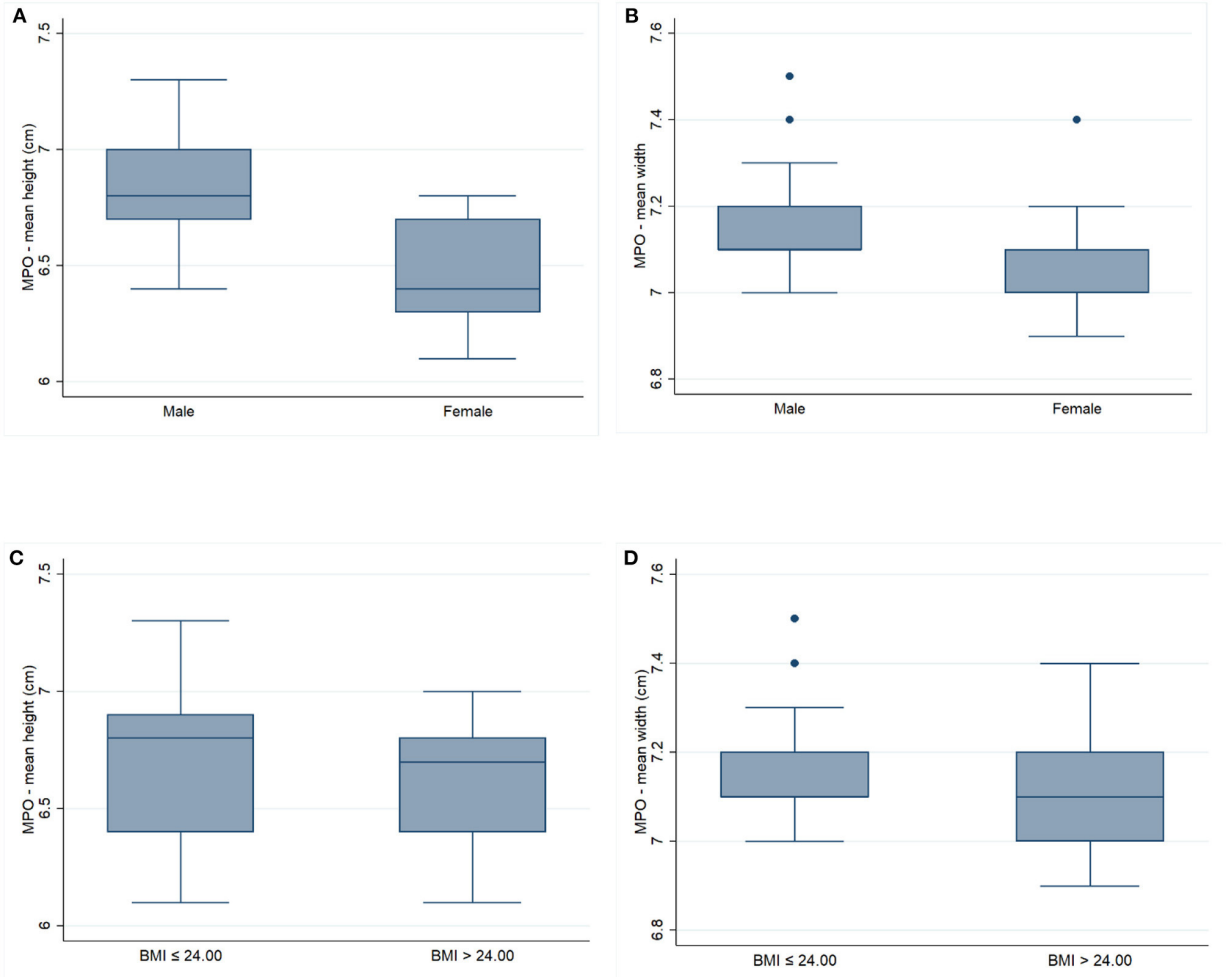
The graph demonstrates the myopectineal orifice according to sex and body mass index; **(A)** mean MPO height in sex; **(B)** mean MPO width in sex; **(C)** mean MPO height in BMI; **(D)** mean MPO width in BMI.

The mean MPO width and height were 7.13 ± 0.14 cm and 6.66 ± 0.32 cm, respectively. The difference of the mean MPO width and height according to sex and BMI (BMI <24 and > 24) is shown in Table 2 and Figure 3.

**Table 2:** The dimension of the myopectineal orifice.

	**Male**	**Female**	***p*-value**
MPO in width	7.16 ± 0.14	7.09 ± 0.12	0.13
MPO in height	6.83 ± 0.27	6.45 ± 0.24	0.0003
	**BMI** **>24.00**	**BMI** **≤24.00**	
MPO in width	7.15 ± 0.14	7.10 ± 0.13	0.36
MPO in height	6.69 ± 0.36	6.60 ± 0.26	0.47

From Monte-Carlo simulations, the mean MPO area was 37.26 ± 0.027 cm^2^. Wolloscheck T. reported the MPO dimension in the cadaveric study and regarding cadaveric shrinkage about 3% compared with a fresh cadaver, or the mean MPO will be increasing to 39.56 ± 0.029 cm^2^ (5). The mesh size that is considered adequate for hernia repair is 10 cm × 15 cm (150 cm^2^). Given the mesh shrinkage of 4.2–46% (6–10), the mesh area was varied from 81 to 143.7 cm^2^. Compared to the size of the MPO, it was found that the mesh size still achieved complete MPO coverage.

## Discussion

Inguinal hernia is the most common problem in general surgery. The mesh-based repair was usually performed in both open and laparoscopic repairs as recommended by the international guideline for groin hernia management (2). The good inguinal hernia repair outcome consists of multiple factors, such as understating the anatomy, optimizing the surgical material, and right surgical techniques (11–14).

Myopectineal orifice (MPO), which was described by Dr. Henri Fruchaud in 1956, is a well-defined weak area in the lower anterior abdomen that most frequently occurs in an inguinal hernia. The MPO boundary consisted of the lateral boundary as the iliopsoas muscle; medial boundary as the rectus sheath and rectus abdominis muscle, superiorly as the arching fibers of the transversus abdominis and internal oblique muscle and tendons; and the interior boundary as the iliopectineal line and Cooper's ligament and Pecten pubis (5, 15). Understanding and identifying the MPO's delineation are essential for surgeons in surgical repair nowadays. The inguinal anatomy should be identified during the surgical approach, both in open and laparoscopic techniques. The hernia sac should be dissected from the MPO, and the prosthetic mesh should cover the entire MPO and extend 3–5 cm beyond the MPO's boundaries to prevent a mesh migration, resulting in recurrence.

The main result of our study, MPO measurements in a Thai cadaver revealed that the dimensions were equal to the mean MPO width and height of 7.13 ± 0.14 cm and 6.66 ± 0.32 cm, respectively. The mean MPO area, using elliptical area equation, is 37.26 ± 0.027 cm^2^. One of the considerations in determining the optimal mesh size for hernia patients is the MPO dimension. Through systematic searching, our results were similar to those of Wolloscheck T. who reported the MPO dimension in the cadaveric study with a mean width of ~7.8 ± 3 cm and a mean height of 6.5 ± 1.9 cm (5). While the mean MPO area measurement was different from the Ndung'u BM study that was calculated with a trapezoid pattern and the mean area was 7 ± 1.29 cm^2^ (16). Because of the curvature of each muscle layer, we selected an oval shape that would better cover the MPO region. The Wolloscheck T. and Ndung'u BM report was measured in Germany and Kenya, respectively. Even though ethnic differences may affect the MPO dimension, our study did not indicate substantial differences.

We chose to measure in cadaver because it allowed us to utilize the proper measurement tool and to measure in all dimensions with consistency. However, the disadvantages of the cadaveric study were that there was no actual hernia and cadaveric tissue would shrink from formalin fixation. We solved the problem by performing a Monte-Carlo Simulation, which creates a simulation dataset by sampling available data repeatedly. It has the advantage that the simulation data we collected is similar to a probability analysis or the chance of this data set being available in the future, allowing us to do a more precise analysis. As for tissue shrinkage, Wolloscheck T's report states that cadavers have 3% shrinkage. Therefore, we have increased the MPO area from the original measured value by 3%.

The mesh material (polypropylene or polyester) and design (flat or anatomical mesh) have also contributed to clinical practice. However, its size continues to be a critical issue that remains controversial (17). Similarly, the mesh has shrinkage, which is reported differently: Harsløf, studied in Physiomesh in various mesh fixation types, has a shrinkage ranging from 17.7 to 35.7% (6). Kuehnert's study evaluated polyvinylidenfluoride (PVDF) meshes in an animal study and demonstrated 30% shrinkage in 30 days (7). Silvestre's study found the percentage of shrinkage was 7.8% for heavyweight mesh and 4.2% for lightweight mesh (8). Inguinal mesh size of 10 cm × 15 cm is usually available in the market; however, the ideal mesh size recommended to cover the MPO ranges from 3 in × 3 in to 3 in × 6 in (7.5 cm × 7.5 cm to 7.5 cm × 15 cm) (18). Therefore, we performed a Monte-Carlo simulation of the mesh size 10 cm × 15 cm using a worsened percentage of shrinkage as compared to the MPO area, or the shrunk mesh area was 81–143.7 cm^2^, that appropriate with MPO area in our study.

## Conclusions

A mesh size of 10 cm × 15 cm is an appropriate size to cover the MPO among Thais.

## Data Availability Statement

The raw data supporting the conclusions of this article will be made available by the authors, without undue reservation.

## Author Contributions

AT, SL, and ST are the principal investigators who contributed to the concept, data collection, analysis, and manuscript writing. TR performed the data collection. WK performed the manuscript review. All authors contributed to the article and approved the submitted version.

## Funding

This study was funded by a grant from the Faculty of Medicine Vajira Hospital, Navamindradhiraj University (Grant Number 034/2558).

## Conflict of Interest

The authors declare that the research was conducted in the absence of any commercial or financial relationships that could be construed as a potential conflict of interest.

## Publisher's Note

All claims expressed in this article are solely those of the authors and do not necessarily represent those of their affiliated organizations, or those of the publisher, the editors and the reviewers. Any product that may be evaluated in this article, or claim that may be made by its manufacturer, is not guaranteed or endorsed by the publisher.
